# Carboplatin, ifosfamide and etoposide with mid-course vincristine and thoracic radiotherapy for 'limited' stage small cell carcinoma of the bronchus.

**DOI:** 10.1038/bjc.1989.228

**Published:** 1989-07

**Authors:** N. Thatcher, M. Lind, R. Stout, C. Payne, K. B. Carroll, C. Campbell, H. Moussalli

**Affiliations:** CRC Department of Medical Oncology, Christie Hospital and Holt Radium Institute, Manchester, UK.

## Abstract

Forty-two patients with small cell lung cancer were treated with a combination of carboplatin, ifosfamide and etoposide. Vincristine was given on day 14 of each course, the courses being repeated every 28 days for a maximum of six. Thoracic radiotherapy was given 4 weeks after the last course of chemotherapy but no prophylactic cranial radiotherapy was administered. Thirty patients had clinically limited state disease, the remaining patients having contralateral neck lymphadenopathy and/or pleural effusions. Elevated enzyme levels (alkaline phosphatase, LDH, ALT, GGT) were noted in 69% of patients. Twenty-four patients (57%) achieved a complete response (CR) when assessed one month after the end of treatment. A further 21% of patients had a partial response (PR). Median duration of CR was 14 months and of PR 8 months. Cerebral metastases were the sole site of relapse in 13% of the CR patients. Myelosuppression was severe with a median nadir of neutropenia of 0.2 x 10(9) cells 1-1. However, 74% of the patient group received all six courses of chemotherapy and only 16 courses (7%) were delayed because of toxicity. There were three deaths associated with treatment-related neutropenia. The median survival of the total group was 14 months, with an actuarial 2 year survival of 37% and a minimum follow-up of 18 months. [A recent analysis, March 1989, demonstrated a 33%, 2 year actual survival.]


Br. J. Cancer (1989), 60, 98-101 © The Macmillan Press Ltd., 1989

### Carboplatin, ifosfamide and etoposide with mid-course vincristine and thoracic radiotherapy for 'limited' stage small cell carcinoma of the bronchus

N. Thatcher[1,4], M. Lind[1], R. Stout[2], C. Payne[3], K.B. Carroll[4], C. Campbell[4] & H. Moussalli[4]

[1]*CRC Department of Medical Oncology and* [2]*Department of Radiotherapy, Christie Hospital and Holt Radium Institute;* [3]*Department of Medicine, Tameside General Hospital, Ashton; and* [4]*Department of Thoracic Medicine and Surgery, Wythenshawe Hospital, Manchester, UK.*

**Summary** Forty-two patients with small cell lung cancer were treated with a combination of carboplatin, ifosfamide and etoposide. Vincristine was given on day 14 of each course, the courses being repeated every 28 days for a maximum of six. Thoracic radiotherapy was given 4 weeks after the last course of chemotherapy but no prophylactic cranial radiotherapy was administered. Thirty patients had clinically limited stage disease, the remaining patients having contralateral neck lymphadenopathy and/or pleural effusions. Elevated enzyme levels (alkaline phosphatase, LDH, ALT, GGT) were noted in 69% of patients. Twenty-four patients (57%) achieved a complete response (CR) when assessed one month after the end of treatment. A further 21% of patients had a partial response (PR). Median duration of CR was 14 months and of PR 8 months. Cerebral metastases were the sole site of relapse in 13% of the CR patients. Myelosuppression was severe with a median nadir of neutropenia of $0.2 \times 10^9$ cells $l^{-1}$. However, 74% of the patient group received all six courses of chemotherapy and only 16 courses (7%) were delayed because of toxicity. There were three deaths associated with treatment-related neutropenia. The median survival of the total group was 14 months, with an actuarial 2 year survival of 37% and a minimum follow-up of 18 months. [A recent analysis, March 1989, demonstrated a 33%, 2 year **actual** survival.]

A number of new agents active against small cell lung cancer are now available. Carboplatin, unlike cisplatin the parent analogue, has several advantages. Carboplatin is less emetic and is without significant nephrotoxicity, neurotoxicity or ototoxicity (Calvert *et al.*, 1982; Smith *et al.*, 1985; Wiltshaw, 1985). Carboplatin has high activity as a single agent in small cell lung cancer with a response rate of 60% and 82% when used with etoposide in previously untreated patients (Smith *et al.*, 1985, 1987). Myelosuppression was the main toxicity but treatment was otherwise well tolerated. Ifosfamide (an isomer of cyclophosphamide) and etoposide with thoracic radiotherapy has given an overall response rate of 97% without severe toxicity, and a median survival of 11 months in limited stage patients (Thatcher *et al.*, 1987). The *actual* 2-year survival (from a more recent analysis) in the above group of patients is 22%. The myelosuppressive carboplatin was therefore combined with the ifosfamide, etoposide and thoracic radiotherapy. Vincristine was also given at mid-course to help prevent relapse between chemotherapy treatments.

### Patients and methods

Forty-two patients with histologically proven small cell lung cancer were treated between August 1985 and May 1986. Twenty-eight patients were men and 14 were women. The median age was 54 years with a range of 38–70 years. Histology was obtained by bronchoscopic examination in 79% of patients. In other patients node biopsy or thoracotomy was performed. Thirty patients had hilar and/or mediastinal lymphadenopathy as the only obvious site of tumour and were of limited stage (LS), defined as tumour confined to one hemithorax, the mediastinum and ipsilateral supraclavicular lymph nodes. The other 12 patients although having more extensive disease (ES) (see Table I) were considered to be of a good prognostic group as defined by previous multivariant analysis (Cerny *et al.*, 1987). In nine patients (21%) the Karnofsky performance status was good, i.e. >80, and in seven patients (17%) the score was reduced to 50 or less (Karnofsky *et al.*, 1948).

### *Treatment regimen*

Carboplatin was given at $300\,mg\,m^{-2}$ as a 1 h i.v. infusion in 500 ml of 5% dextrose on day 1. Etoposide was administered at $120\,mg\,m^{-2}$ i.v. on days 1 and 2 and $240\,mg\,m^{-2}$ orally on day 3. Ifosfamide $5\,g\,m^{-2}$ with the same dose of mesna was given immediately after the carboplatin as a 24 h, 2 l, N

**Table I** Pretreatment clinical features of the 42 patients

| | *LS (30)* | *ES (12)* |
|---|---|---|
| Interval from symptoms to diagnosis | | |
| <1-3 months | 16 | 8 |
| 3–6 months | 13 | 3 |
| >6 months | 1 | 1 |
| Interval from diagnosis to treatment | | |
| <1 month | 20 | 12 |
| 1–2 months | 9 | – |
| >2 months | 1 | – |
| Weight loss (>10% over 6 months) | 14 | 8 |
| Superior vena caval obstruction/stridor | 4 | 2 |
| Lymphadenopathy | | |
| Hilar | 30 | 9 |
| Mediastinal | 20 | 6 |
| Ipsilateral SCF | – | 3 |
| Contralatral/cervical | – | 4 |
| Pleural effusion | – | 9 |
| Elevated enzymes | | |
| Alkaline phosphatase | 7 | 5 |
| Lactate dehydrogenase | 8 | 5 |
| ALT/GGT | 14 | 4 |
| Normal enzyme levels | 9 | 4 |
| Karnofsky performance median (and range) | 60 (40–90) | 60 (40–80) |

LS, limited stage; ES, extensive stage.

Correspondence: N. Thatcher, Department of Medical Oncology, Christie Hospital and Holt Radium Institute, Manchester M20 9BX, UK.
Received 27 October 1988, and accepted in revised form 3 March 1989.

saline i.v. infusion. A further infusion (1 l) of Mesna, $3\,gm^{-2}$, was continued over the subsequent 12 h. Vincristine $0.5\,mg\,m^{-2}$ i.v. was given on day 14 (mid-course). Treatment was repeated every 4 weeks for a maximum of six courses. No dose reductions were undertaken but treatment was deferred to allow blood count recovery if platelets were less than $100,000\,\mu l^{-1}$ or white count was less than 3,000 cells $\mu l^{-1}$. Lorazepam 1 mg i.v. was prescribed and if required metoclopramide, to control nausea and vomiting.

Thoracic megavoltage radiotherapy was given to 36 patients 4–5 weeks after the last course of chemotherapy. The post-chemotherapy volume was irradiated with a margin of at least 2 cm around any residual disease, to include the mediastnum and supraclavicular fossa (SCF) if disease was identified on presentation. In patients with complete radiological response, the field was centred on the site of the original thoracic tumour. Two treatment schemes were employed: (a) 75% of patients received a single fraction of 12.5 Gy using a 360° rotation technique; the median field size was 10 × 9 × 9 cm; (b) the other 25% in whom rotation was not technically feasible received 27.5 Gy midline dose in eight fractions over 10 days using a parallel opposed pair with a median field size of 12 × 9 cm. Two other patients because of extensive disease received wide field thoracic radiotherapy. The remaining four patients were not irradiated, having died before the completion of chemotherapy. A separate matched single field of 12.5 Gy in one fraction was prescribed for SCF disease.

*Investigations before and during treatment*

All patients had a complete clinical examination with chest radiograph, full blood count and routine biochemistry including hepatic enzymes before each treatment together with evaluation of the Karnofsky and Respiratory scores (Karnofsky *et al.*, 1948; Medical Research Council Lung Cancer Working Party, 1979). Bone marrow examination, radionucleide and ultrasound scans were performed before the first chemotherapy course to confirm clinical or biochemical abnormalities suggestive of metastatic disease. If these scan or marrow examinations indicated metastatic disease the patient was not entered into the study. Patients with brain metastases, with Karnofsky scores of 30 or less, those aged over 70 years or more and patients previously treated with chemotherapy and radiotherapy were ineligible for the study. Repeat bronchoscopy was also requested 4–6 weeks after thoracic radiotherapy.

Toxicity was graded according to standard WHO criteria (Miller *et al.*, 1981) except for nausea and vomiting which was scored according to Smith *et al.* (1987). The nadir blood count was taken from weekly counts after chemotherapy. If disease progressed the treatment protocol was discontinued and symptomatic measures were instituted.

*Follow-up*

At the end of treatment patients were seen at monthly intervals for 4 months then 3-monthly for a year and every 6 months thereafter. Assessment for objective response was undertaken at first follow-up (1 month after the end of treatment) and was determined by standard criteria (Miller *et al.*, 1981).

### Results

*Response and survival*

Twenty-four patients or 57% (95% confidence limits, 42–72%) of the total patient group were classified as in complete response one month after the end of treatment (Table II). One other patient who had had a pneumonectomy was not radiologically evaluable for response but was free of tumour at rebronchoscopy at the end of treatment.

Another nine patients (21%, 95% confidence limits, 9–33%) had a partial response and in five patients there was progressive tumour. The remaining three patients died of probable infection and leucopenia. The median duration of partial response was 8 months (range 5–13 months) and of complete response 14 months (range 8–26+ months). Of the patients who eventually responded, 81% had shown a response with the first chemotherapy course and the remainder by the third course. Eighteen patients in clinical and radiological complete remission agreed to be rebronchoscoped and in 17 no evidence of tumour was found.

The median survival of all 42 patients was 14 months (range 1–26+ months, see Figure 1). The actuarial 2-year survival is 37% (95% confidence limits for 24 months are 21.5–50.1 by the Greenwood formula with the Kalbfleisch Prentice adjustment). The median survival of the 30 limited stage patients was again 14 months with the same range and two year actuarial survival. The median survival for patients undergoing partial response was 11 months (range 6–14 months) and for non-responders 1 month (range <1–7 months). The median survival for complete responders has not yet been reached. Of the five patients alive at two or more years, four were of limited stage with no supraclavicular lymphadenopathy and the other had a pleural effusion: the median Karnofsky score was 70 (range 50–80) and the LDH was raised in three patients including also one patient with an elevated alkaline phosphatase.

**Table II** Response, relapse, progression status and stage

| | NR | | PR | | CR | | NE |
|---|---|---|---|---|---|---|---|
| | LS | ES | LS | ES | LS | ES | LS |
| Patient numbers | 6 | 2 | 7 | 2 | 16 | 8 | 1 |
| Alive | | | | | | | |
| No relapse/progression | – | – | – | – | 8 | 4 | 1 |
| Relapse/progression | – | – | – | – | 2 | – | – |
| Dead | | | | | | | |
| Not of lung carcinoma | 2 | 1 | 1 | – | – | – | – |
| Of lung carcinoma[a] | 4 | 1 | 6 | 2 | 6 | 4 | – |

LS, limited stage; ES, extensive stage; NR, non-responder; PR, CR, partial, complete responders; NE, not evaluable for response.
[a]Includes relapsed patients and patients with progressive tumour.

**Figure 1** Survival of total patient group ($n$ = 42; ——) and of the Limited Stage group ($n$ = 30; – – –).

### *Relapse*

Twenty patients have developed recurrent tumour following response (Table II). Of the 12 patients who had a complete response, one patient had local relapse only within or adjacent to the irradiated zone, a further five patients had distant relapse only and the remaining six patients had a combination of both local and distant relapse. There were eight patients with a partial response who relapsed, one locally only, six with distant metastases and the remaining patient both locally and distantly. Three patients in the complete responder group developed brain metastases as the apparent sole site of recurrence. The pattern of relapse is given in further detail in Table III. Six patients recieved radiotherapy for relapse and five patients further chemotherapy with an adriamycin regimen.

### *Toxicity*

Thirty-one patients (74%) received all six courses of chemotherapy, six patients four courses and two patients five courses. One patient received three courses and two patients one course only. A total of 225 courses (89%) of the possible maximum 252 courses was therefore administered. Fifteen courses were delayed by 1 week and one course by 2 weeks because of toxicity. The tendency for an increase in haematological toxicity with increasing number of courses can be seen in Table IV. On 50 occasions blood transfusions were given accounting for 167 units of packed cells. Severe thrombocytopenia requiring platelet transfusions occurrèd on 11 occasions. On 13 occasions severe or life-threatening leucopenia (WBC $< 1.9 \times 10^9$ cells $l^{-1}$) was noted 4 weeks after a chemotherapy course. The marked neutropenia with a median of $0.2 \times 10^6$ neutrophils $l^{-1}$ occurring 2 weeks after chemotherapy is shown in Figure 2. On three occasions infection was thought to have contributed to treatment related death. One patient who was hypertensive developed severe renal impairment following the first treatment. No other renal or urothelial toxicity was noted although partial alopecia was universal. On only seven courses was there severe nausea and vomiting requiring anti-emetics for more than 24 h and on 47 courses nausea and vomiting was of moderate grade. Transient oesophagitis occurred in the majority of patients but there was no unusual toxicity from the radiotherapy. The overall improvement in Karnofsky and Respiratory score with treatment can be seen in Table V.

**Table III** Sites of distant relapse

| | PR | CR |
|---|---|---|
| Nodes | | |
| SCF | 1 | – |
| Upper abdomen | – | 2 (1) |
| Contralateral lung | 1 | – |
| Brain | 7 (4) | 8 (3) |
| Liver | 1 | 3 (1) |
| Bone | – | – |
| Skin | – | 1 |

SCF, supraclavicular fossa. Patients without local relapse in primary tumour area but with single site of metastasis indicated in parentheses.

### Discussion

A combination of carboplatin, ifosfamide and etoposide with mid-course vincristine is an effective combination for small cell lung cancer. The objective response rate was 78% for the total patient group and 90% for the 38 patients evaluable for response at the end of treatment. The response rate is similar to that found with carboplatin and etoposide used as first-line treatment described by Smith *et al.* and our earlier ifosfamide and etoposide combination (Smith *et al.*, 1987; Thatcher *et al.*, 1987). The median survival of 14 months is better than the 9.5 months and 11 months previously described with the two agent combinations in similar patient groups (Smith *et al.*, 1987; Thatcher *et al.*, 1987). Furthermore, the improved median survival and actuarial 2 years survival of 37% was observed in a patient group which included pleural effusions, contralateral lymph nodes and elevated hepatic enzymes.

Myelosuppression was a serious problem and three patients died (with no evidence of tumour), probably from

**Figure 2** Median neutrophil count during chemotherapy.

**Table V** Change in Karnofsky performance (KP) score and respiratory assessment score (RA) with treatment

| | Pretreatment | Score after CT course 2 | Score after CT course 4 | Post-treatment |
|---|---|---|---|---|
| *KP score* | | | | |
| <50[a] | 2% | 5% | 7% | 14% |
| 50–70 | 77% | 17% | 10% | 7% |
| 80–100 | 21% | 78% | 83% | 79% |
| *RA score* | | | | |
| 1,2 | 21% | 83% | 84% | 78% |
| 3,4 | 62% | 12% | 9% | 7% |
| 5[a] | 17% | 5% | 7% | 15% |

[a]Includes patients dying.

Grade 1,2 climb hills, stairs, walk any distance on the flat at normal pace, without dyspnoea; grade 3,4 walk more than 100 yards at own speed without dyspnoea, dyspnoea on walking 100 yards or less. Grade 5, dyspnoea on mild exertion, e.g. undressing.

**Table IV** Haematological toxicity – worst grade based on nadir counts

| Course no. | 1 | | | 2 | | | 3 | | | 4 | | | 5 | | | 6 | | |
|---|---|---|---|---|---|---|---|---|---|---|---|---|---|---|---|---|---|---|
| | Hb | WBC | Plat | Hb | WBC | Plat | Hb | WBC | Plat | Hb | WBC | Plat | Hb | WBC | Plat | Hb | WBC | Plat |
| Grade 0 | 16 | 1 | 12 | 9 | 2 | 15 | 6 | 1 | 12 | 4 | – | 4 | 2 | 1 | 3 | 1 | 1 | 5 |
| 1 | 15 | – | 3 | 11 | 1 | 3 | 11 | 1 | 5 | 13 | – | 4 | 8 | – | – | 9 | – | 2 |
| 2 | 10 | 10 | 13 | 18 | 6 | 6 | 14 | 8 | 5 | 12 | 5 | 7 | 18 | 2 | 7 | 18 | 4 | 5 |
| 3 | 1 | 25 | 10 | 3 | 22 | 10 | 8 | 19 | 7 | 10 | 16 | 11 | 4 | 15 | 7 | 2 | 13 | 6 |
| 4 | – | 6 | 4 | 0 | 10 | 7 | 1 | 11 | 11 | – | 18 | 13 | 1 | 15 | 16 | – | 12 | 12 |
| NA/NK | – | – | – | 1 | 1 | 1 | 2 | 2 | 2 | 3 | 3 | 3 | 9 | 9 | 9 | 12 | 12 | 12 |

NA/NK, not applicable or not known.

infection and neutropenia. However, the myelosuppression was transient and only 7% of all courses were delayed due to toxicity, and no dosage reductions were made. There was no evidence of nephrotoxicity except in one patient and treatment had to be discontinued due to renal failure following the first course. In no other patient was elevation of serum creatinine noted, and lack of nephrotoxicity compared with cisplatin was confirmed.

The incorporation of vincristine between treatment courses may have prevented the between course relapse described by Smith *et al.* (1987) and brain metastases may have been reduced by prophylactic cranial irradiation. Future studies using haemopoietic stimulating factors may well reduce myelosuppression and allow further development of chemotherapeutic strategies.

### References

CALVERT, A.H., HARLAND, S.J., NEWELL, D.R. and 9 others (1982). Early clinical studies with cis-diamine I,I-cyclobutane dicarboxylate platinum II. *Cancer Chemother. Pharmacol.*, **9**, 140.

CERNY, T., BLAIR, V., ANDERSON, H., BRAMWELL, V. & THATCHER, N. (1987). Pre-treatment prognostic factors and scoring system in 407 small-cell lung cancer patients. *Int. J. Cancer*, **39**, 146.

KARNOFSKY, D.A., ABLEMAN, W.H., CRAVEN, L.F. & BURCHENAL, J.H. (1948). The use of nitrogen mustards in the palliative treatment of carcinoma. *Cancer*, **1**, 634.

MEDICAL RESEARCH COUNCIL LUNG CANCER WORKING PARTY (1979). Radiotherapy alone or with chemotherapy in the treatment of small cell carcinomas of the lung. *Br. J. Cancer*, **40**, 1.

MILLER, A.B., HOOGSTRATEN, B. & STAQUET, M. (1981). Reporting results of cancer treatment. *Cancer*, **47**, 207.

SMITH, I.E., EVANS, B.D., GORE, M.E., REPETTO, V.L., YARNOLD, J.R. & FORD, H.T. (1987). Carboplatin (paraplatin; JM8) and etoposide (VP-16) as first-line combination therapy for small cell lung cancer. *J. Clin. Oncol.*, **5**, 185.

SMITH, I.E., HARLAND, S.J., ROBINSON, B.A. and 7 others (1985). Carboplatin: a very active new cisplatin analog in the treatment of small cell lung cancer. *Cancer Treat. Rep.*, **69**, 43.

THATCHER, N., CERNY, T., STOUT, R. and 5 others (1987). Ifosfamide, etoposide and thoracic iradiation therapy in 163 patients with unresectable small cell lung cancer. *Cancer*, **60**, 2382.

WILTSHAW, E. (1985). Ovarian trials at the Royal Marsden. *Cancer Treat. Rev.*, **12**, suppl., 67.


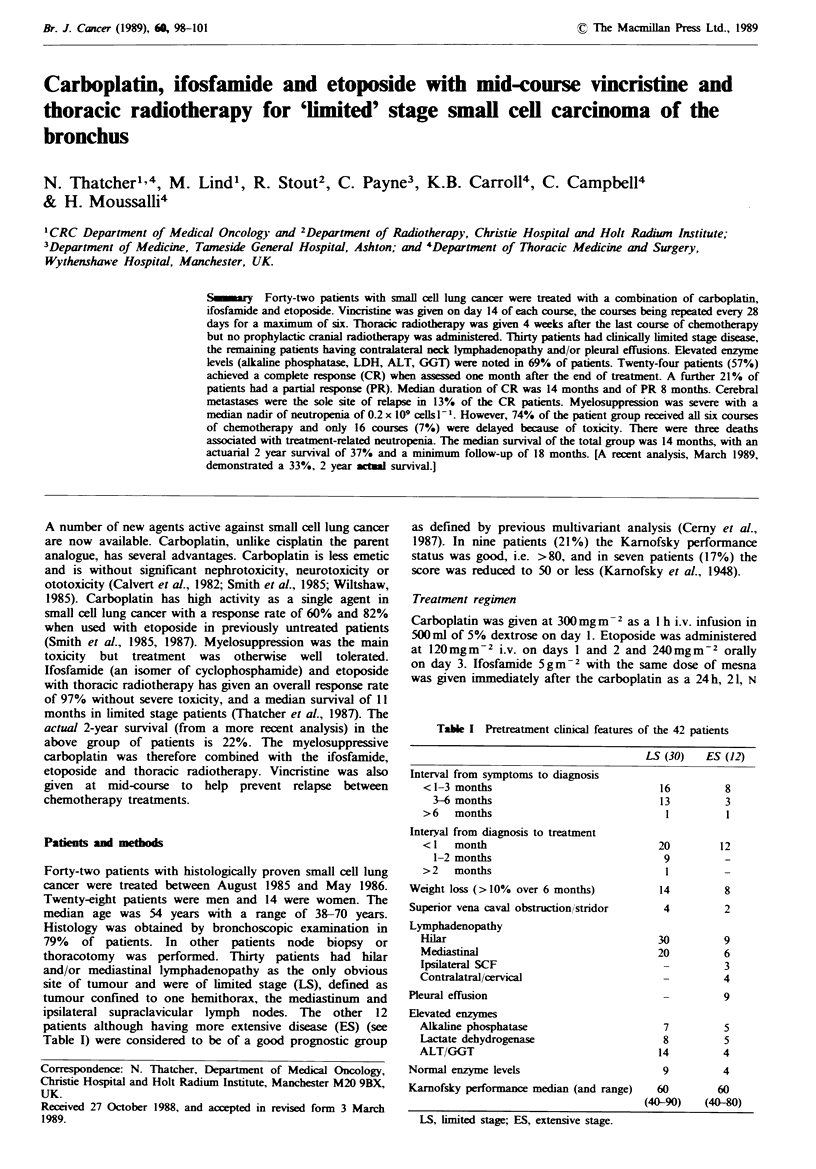

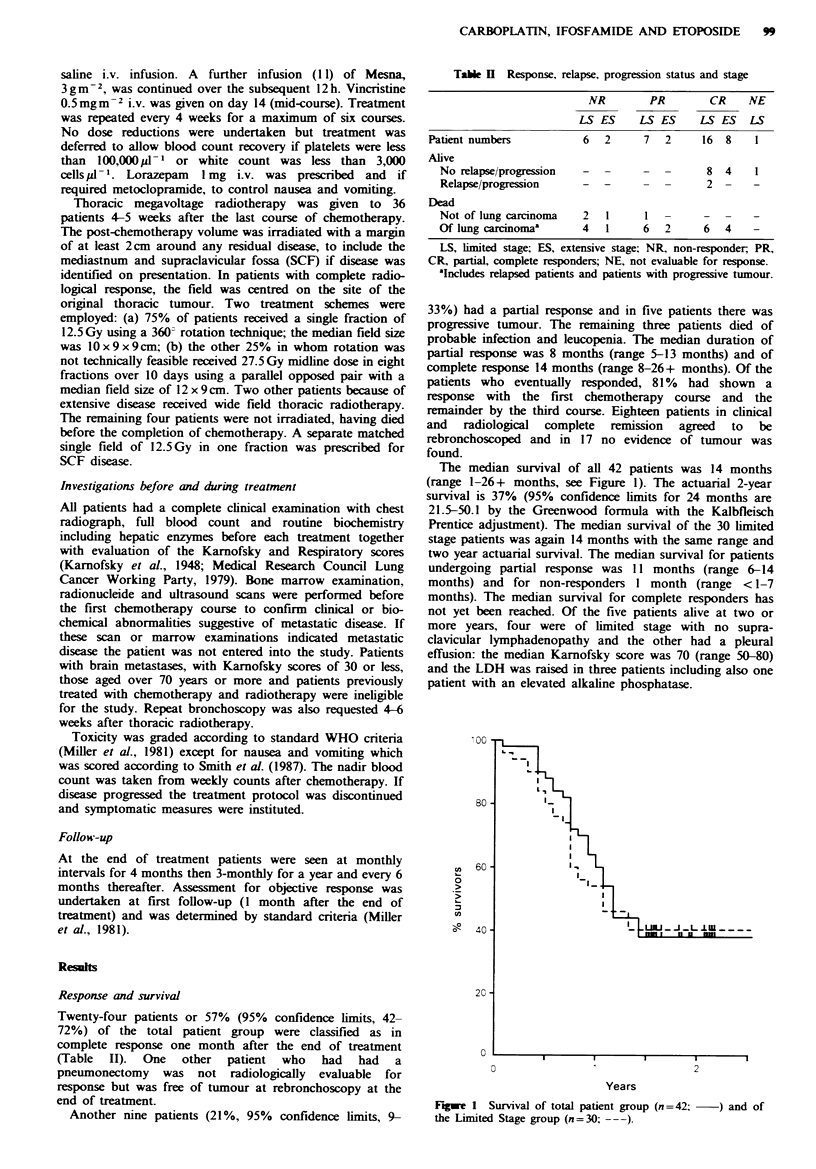

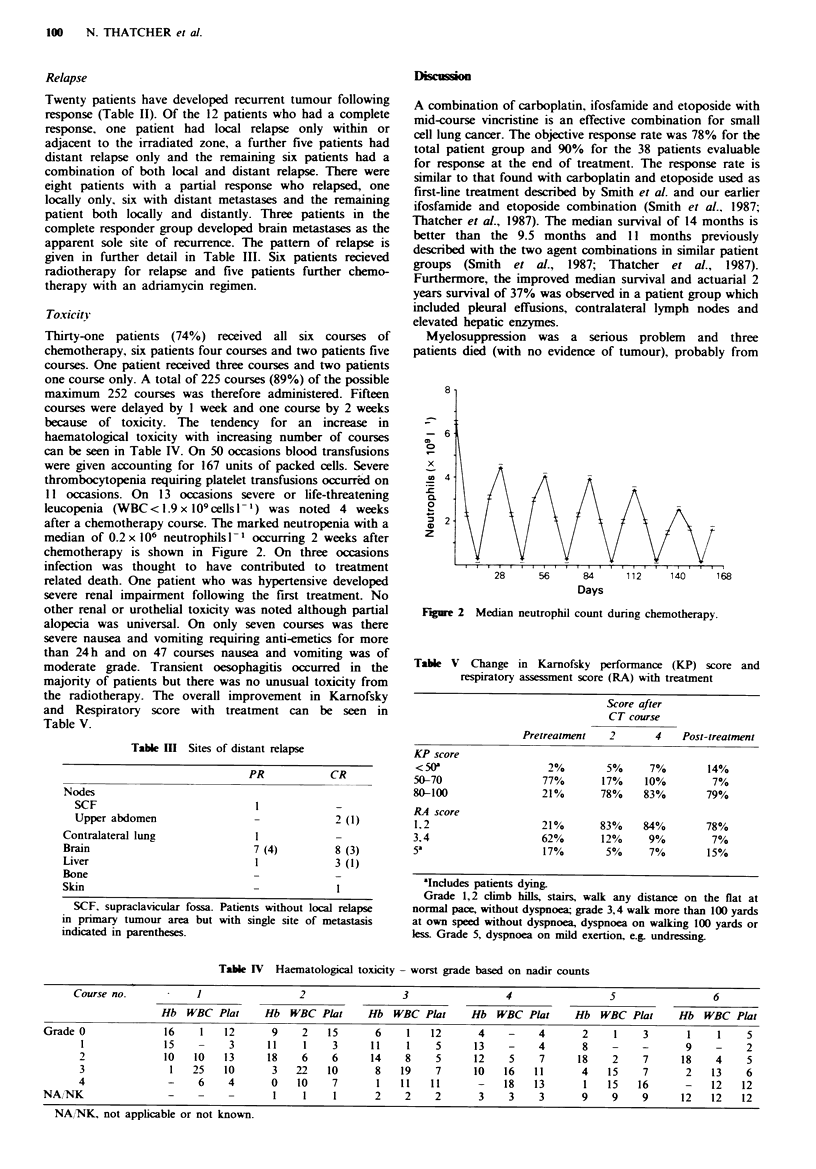

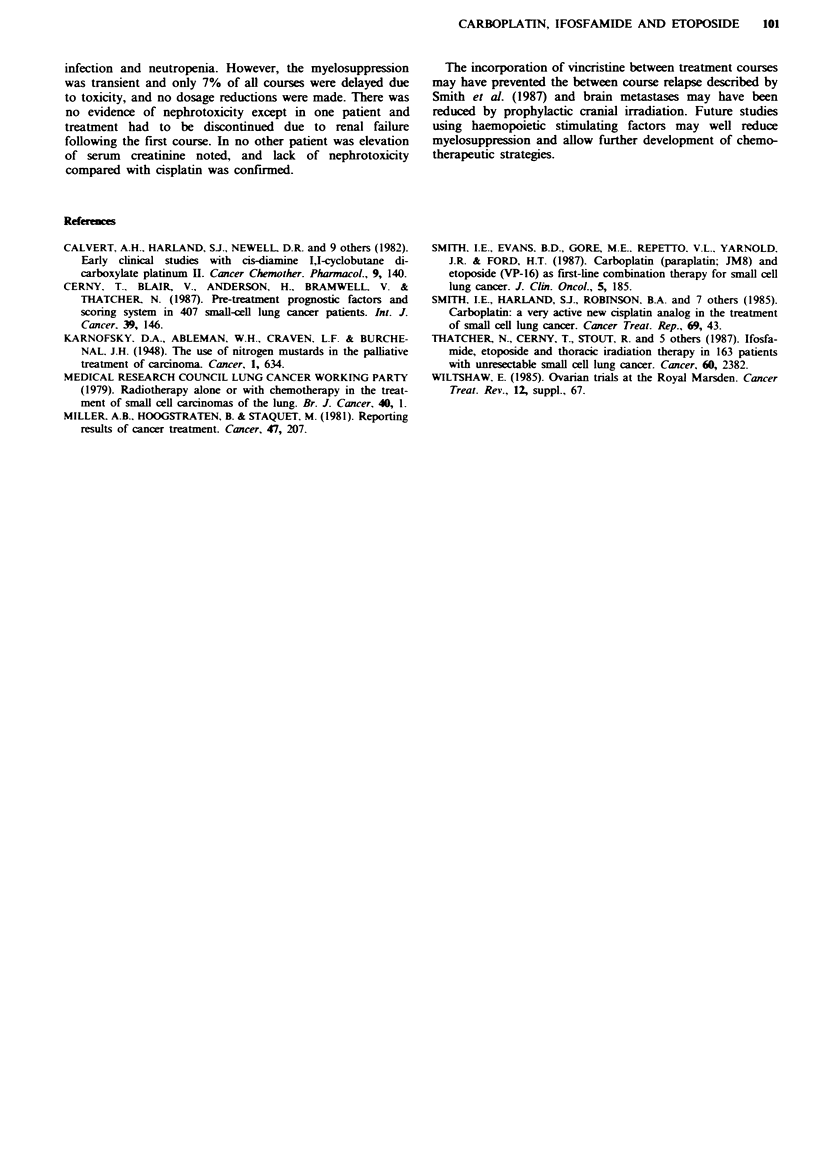


## References

[OCR_00506] Calvert A. H., Harland S. J., Newell D. R., Siddik Z. H., Jones A. C., McElwain T. J., Raju S., Wiltshaw E., Smith I. E., Baker J. M. (1982). Early clinical studies with cis-diammine-1,1-cyclobutane dicarboxylate platinum II.. Cancer Chemother Pharmacol.

[OCR_00510] Cerny T., Blair V., Anderson H., Bramwell V., Thatcher N. (1987). Pretreatment prognostic factors and scoring system in 407 small-cell lung cancer patients.. Int J Cancer.

[OCR_00525] Miller A. B., Hoogstraten B., Staquet M., Winkler A. (1981). Reporting results of cancer treatment.. Cancer.

[OCR_00529] Smith I. E., Evans B. D., Gore M. E., Vincent M. D., Repetto L., Yarnold J. R., Ford H. T. (1987). Carboplatin (Paraplatin; JM8) and etoposide (VP-16) as first-line combination therapy for small-cell lung cancer.. J Clin Oncol.

[OCR_00540] Thatcher N., Cerny T., Stout R., Anderson H., Barber P. V., Wolstenholme R. J., Barnes P., Deiraniya A. (1987). Ifosfamide, etoposide, and thoracic irradiation therapy in 163 patients with unresectable small cell lung cancer.. Cancer.

